# Case Report: Anaphylaxis caused by traditional Chinese medicine in a patient with pollinosis

**DOI:** 10.3389/falgy.2024.1470638

**Published:** 2024-11-01

**Authors:** Zhouxian Pan, Mengyuan Zhan, Qing Wang, Jun Liu, Yu Li, Fan Zhi, Jing Zhang, Jinhe Liu, Kai Guan, Liping Wen

**Affiliations:** ^1^ Department of Allergy, State Key Laboratory of Complex Severe and Rare Diseases, Peking Union Medical College Hospital, Chinese Academy of Medical Sciences and Peking Union Medical College, Beijing, China; ^2^ Allergy Department, Beijing Key Laboratory of Precision Medicine for Diagnosis and Treatment of Allergic Diseases, National Clinical Research Center for Dermatologic and Immunologic Diseases, Peking Union Medical College Hospital, Chinese Academy of Medical Sciences and Peking Union Medical College, Beijing, China; ^3^ Department of Traditional Chinese Medicine, Peking Union Medical College Hospital, Peking Union Medical College, Chinese Academy of Medical Science, Beijing, China

**Keywords:** case report, anaphylaxis, traditional Chinese medicine, Suan Zao Ren, Ziziphus jujuba

## Abstract

This case describes a patient with anaphylaxis caused by traditional Chinese medicine. Skin prick test with the traditional Chinese medicine decoction indicates that he was allergic to Suan Zao Ren. The patient had pollinosis and had never taken Suan Zao Ren before, thus we need to think the possibility of pollen food allergy syndrome. This paper also proposes a procedure for doctors to identify the specific culprit of traditional Chinese medicine decoction.

## Introduction

A 28-year-old male with a history of pollinosis presented to the emergency department in anaphylaxis minutes after taking traditional Chinese medicine decoction. In November 2018, he took traditional Chinese medicine decoction because of insomnia. Minutes within oral intake of the herbal liquid, he developed mucosal edema and dyspnea, without asphyxia, unconscious loss and skin rash on the trunk of limbs. Half an hour after his admission, the dyspnea was aggravated, and he had intensive itchiness all over the body. Blood oxygen fraction was lower than 70% (without oxygen inhalation). The patient refused to wash his stomach and required emetic treatment. After oxygen inhalation, SpO2 recovered to 97% (oxygen inhalation through nasal catheter 2–3l/min). Laboratory tests showed the routine blood test was normal. Blood gas analysis showed the standard oxygen partial pressure was 69 mmHg, the standard partial pressure of carbon dioxide was 38 mmHg, and the level of lactic acid was 2.6 mmol/L (normal range: 0.5–1.6 mmol/L). His symptoms gradually resolved within hours. The patient had a documented history of pollinosis, including allergies to balloonflower and safflower. During peak pollen seasons, he typically presented with concurrent rhinitis and conjunctivitis, consistent with pollinosis.

**Figure 1:**
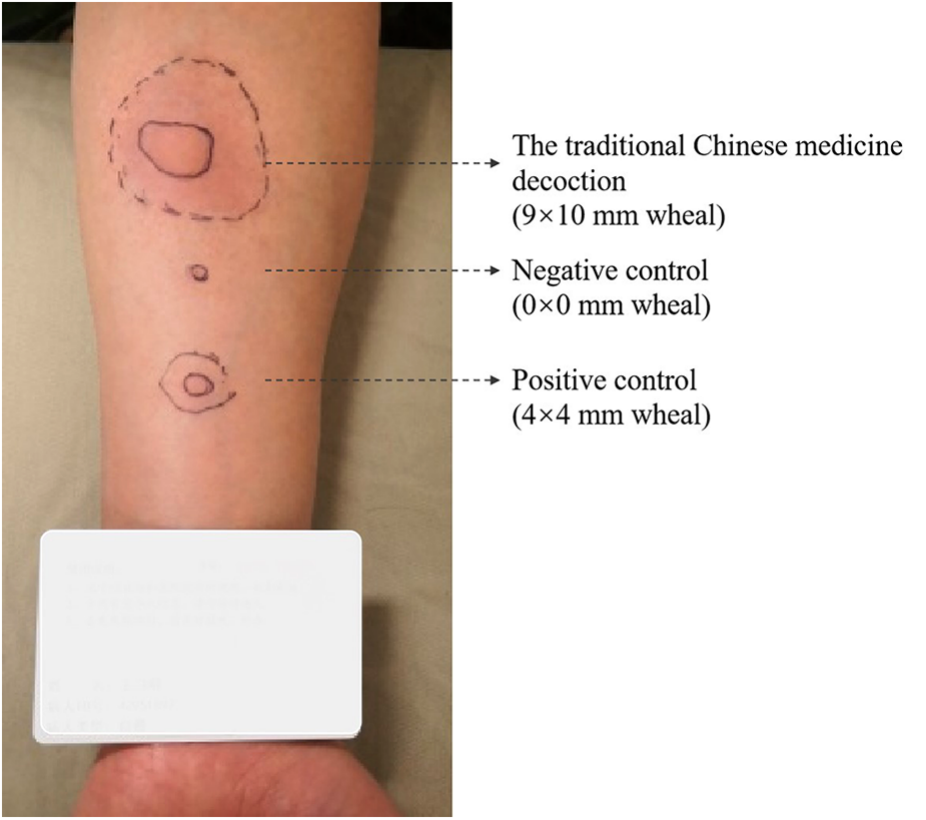
The result of skin prick test with the traditional Chinese medicine decoction (original concentration) (9 × 10 mm wheal). The detailed component of the decoction is shown in Figure 2A.

**Figure 2:**
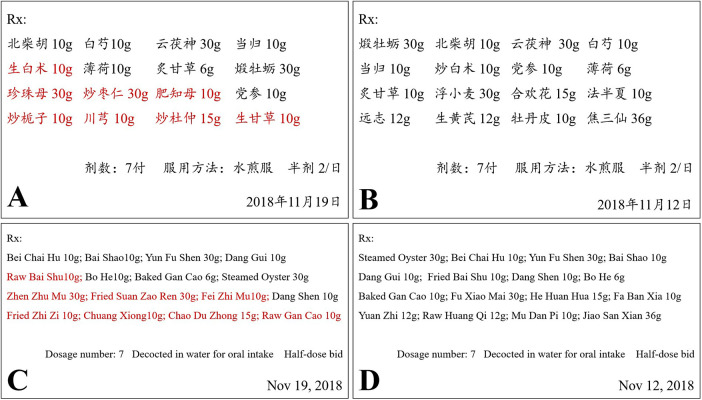
The prescriptions of the traditional Chinese medicine decoction. **(A)** The decoction which caused anaphylaxis. The components in red color were not contained in decoction **(B)**. **(B)** The decoction which did not cause any adverse reactions. **(C)** The translated version of **(A)**. **(D)** The translated version of **(B)**.

**Figure 3:**
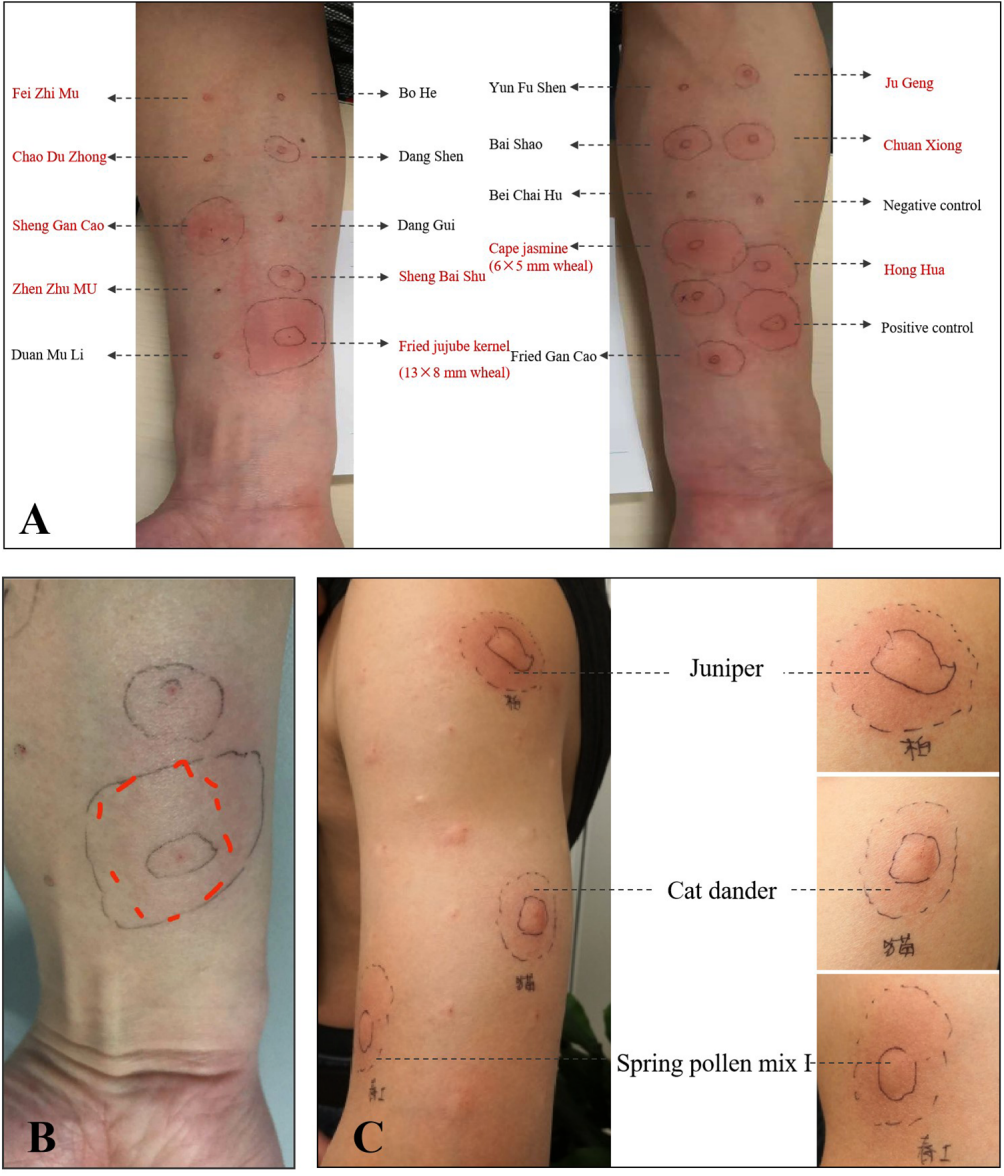
**(A)** The result of the skin prick test for all the components shown in Figure 2A as well as balloon flower and safflower. **(B)** After 6 h, all the skin reactions resolved except for Suan Zao Ren. **(C)** The result of intradermal skin test showed positive reactions to cat dander, Juniper and Spring pollen mix.

Ten days later, he came to our clinic for allergen test. The skin prick test with the traditional Chinese medicine decoction (original concentration) was strongly positive (Figure 1, 9 × 10 mm wheal). He provided the detailed prescription of the traditional Chinese medicine decoction which caused the anaphylaxis (Figures 2A,C). It is noteworthy that one week before the anaphylaxis, he also had taken the traditional Chinese medicine decoction (Figures 2B,D) but had no adverse reactions. We performed skin prick test with the allergen leaching solution of all the components in Figure 2A as well as balloonflower and safflower. The reactions to Fried jujube kernel and Cape jasmine fruit were strongly positive (Figure 3A). After 6 h, all the skin reactions resolved except for Fried jujube kernel (Figure 3B). The patient declined skin prick test of the decoction without fried Suan Zao Ren and Cape jasmine fruit, due to the inconvenience of coming to the hospital again. He also declined oral challenge to Fried Suan Zao Ren, given the severity of his initial reaction. Since the patient had a history of pollinosis, we also performed intradermal skin test on this patient with common inhaled allergens. The result showed positive reactions to cat dander, Juniper and Spring pollen I (Spring Pollen 1 is a mixture that includes five main pollen species: *Cryptomeria fortunei, Cunninghamia lanceolata, Populus Cathayana, Ulmus pumila, Salix matsudana*) (Figure 3C).

It was concluded that this patient had an IgE-medicated anaphylactic reaction to Fried Suan Zao Ren. He was advised to strictly avoid all Fried Suan Zao Ren products and carry an epinephrine autoinjector at all times.

## Discussion

Suan Zao Ren, the seed of Ziziphus jujuba variety spinosa, is an herb commonly used in traditional Chinese medicine. The seeds of Ziziphus jujuba, known as “Suan Zao Ren” and celebrated as “the fruits of life” in China, offer notable benefits for the central nervous system. These seeds are commonly used in traditional Chinese medicine to improve sleep quality, enhance memory, and support learning. Apart from its dietary use, the entire plant, including the seeds, crude leaves, and stem bark, is utilized in ethnopharmacology across various cultures to address a range of ailments from digestive disorders to respiratory issues (1). There are no reported cases of allergy to Suan Zao Ren. The positive skin prick test of this case suggested an IgE-mediated response to the decoction.

Anaphylaxis caused by oral Traditional Chinese medicine is not common. In a report analyzing the data from Beijing pharmacovigilance database, oral traditional Chinese medicine only accounted for 3.5% of all anaphylaxis reports by traditional Chinese medicine (5 of 141), while 135 cases were induced by injection (2). There are reported cases of anaphylaxis caused by Indian jujube (3) and Ginseng (4), which is also a commonly used traditional Chinese medicine. As traditional Chinese medicine have been increasingly used world-wide, it is important to take traditional Chinese medicine into consideration when evaluating a patient with anaphylaxis.

**Figure 4:**
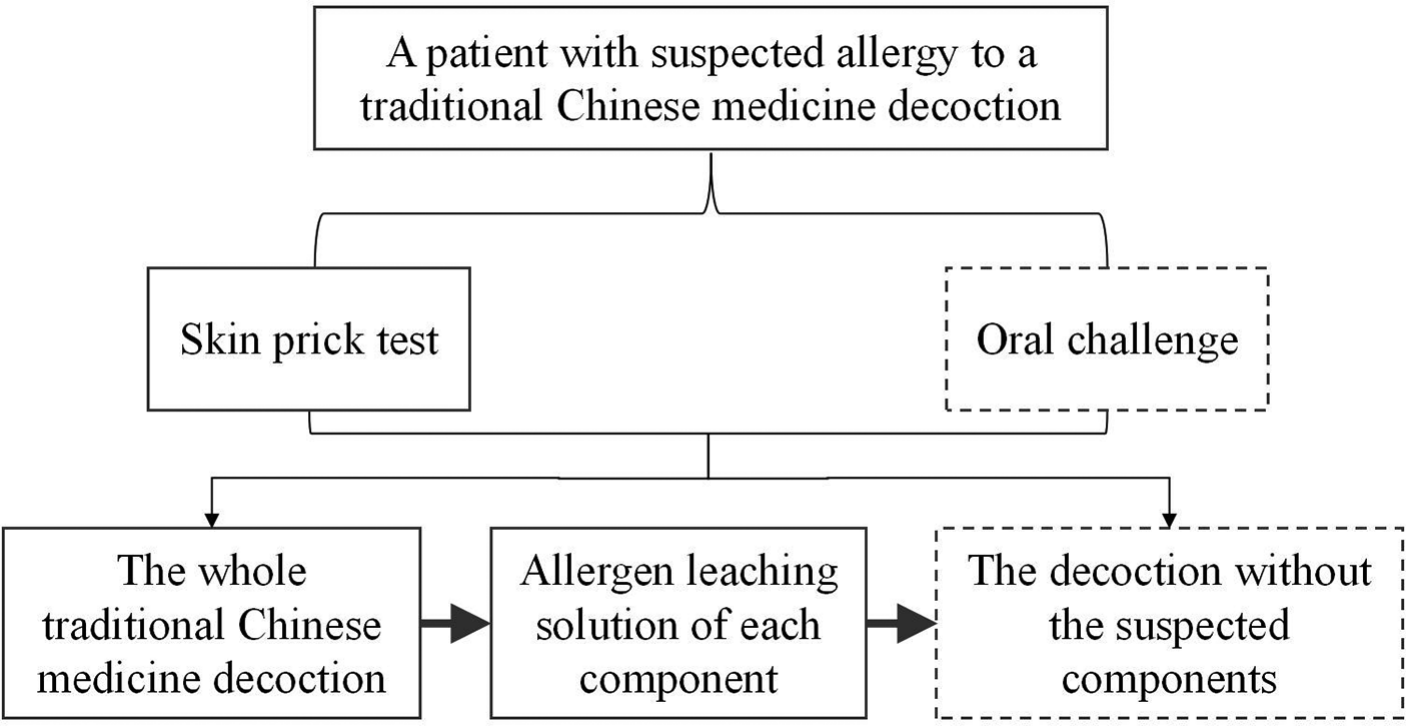
The procedure for doctors to identify the specific culprit of traditional Chinese medicine decoction. In this case, the patient refused the procedures in dotted box.

It is challenging to identify which component of traditional Chinese medicine is the culprit for allergy, because most traditional Chinese medicine are complex mixed formulations. We provide a detailed description of the formula of the decoction which caused anaphylaxis in this patient. In addition, we established a standardized procedure for the diagnosis of traditional Chinese medicine allergy and for identification of the specific culprit (Figure 4). We suggest that the patients with suspected traditional Chinese medicine allergy should be performed the procedure in Figure 4 to look for the cause of allergy. A database of the results of the standardized procedure would be beneficial for the diagnosis of traditional Chinese medicine allergy in the future.

The patient had pollinosis and had never taken Suan Zao Ren before, thus we need to think the possibility of pollen food allergy syndrome (5). It is important to identify the possible allergens in traditional Chinese medicine that have cross-reactivity with pollen allergens.

Given the fact that Suan Zao Ren is widely used, more cases of such allergy may be reported in the future. Further studies investigating the mechanism underlying the symptoms in response to traditional Chinese medicine would be valuable.

Continuing from our discussion on traditional Chinese medicine and its implications for allergic reactions, we turn our focus to the cross-reactivity observed within the Cupressaceae family, particularly involving Juniper and its potential allergenic relationships. Juniper, like other members of this family, has been shown to exhibit cross-reactivity with various fruits through allergenic proteins such as lipid transfer proteins (LTPs), which are also implicated in systemic food allergies and pollinosis (6–10).

In the specific context of our patient, the primary allergen identified was cypress pollen. Previous studies have documented the cross-reactivity between cypress and certain fruits like peach, which was also evident in the referenced study where patients allergic to peach showed reactions to cypress pollen (6–8). These reactions are attributed to the presence of a 15-kDa LTP in cypress pollen, demonstrating cross-reactivity with peach LTP (Pru p 3). Such findings underscore the potential for similar cross-reactive patterns with Juniper, possibly extending to other fruits, including Jujuba (6).

Given the phylogenetic proximity and the shared presence of LTPs within the Cupressaceae family, it is a reasonable hypothesis that Juniper may also demonstrate cross-reactivity with Jujuba. However, to affirm this hypothesis, extensive immunological assays, such as competitive inhibition experiments and component-resolved diagnostics, would be necessary to confirm the presence and cross-reactivity of specific allergens.

## Data Availability

The original contributions presented in the study are included in the article/Supplementary Material, further inquiries can be directed to the corresponding authors.
